# Successful Management of a Huge Pulmonary Hydatid Cyst with Lung-Preserving Surgery

**DOI:** 10.1155/2020/9526406

**Published:** 2020-03-17

**Authors:** Armin Amirian, Bizhan Ziaian, Amirhossein Erfani, Reza Shahriarirad, Keivan Ranjbar

**Affiliations:** ^1^Thoracic and Vascular Surgery Research Center, Shiraz University of Medical Sciences, Shiraz, Iran; ^2^Student Research Committee, Shiraz University of Medical Sciences, Shiraz, Iran

## Abstract

The lung is the second most commonly involved organ in humans by hydatid disease. Management of large pulmonary hydatid cysts is a great challenge for thoracic surgeons. Lung resections should be considered the last choice for huge pulmonary hydatid cysts when the lung expansion is not optimal after cyst removal. Here, we present a case of huge lung hydatid cyst involving the entire right lower lobe which was successfully managed by lung-preserving surgery in which the postoperative course showed gradual resolution of the involved lobe during a one-year follow-up.

## 1. Introduction

After the liver, the lung is considered to be the second most infected part of the body in hydatid disease [1–3]. Sign and symptoms of the patients may vary depending on the size and location of the cyst, ranging from asymptomatic to severe dyspnea, cough, chest tightness, and pain [4, 5]. Even though hospitalization due to human cystic hydatidosis has decreased, some parts of the world such as the Middle East are still considered to be an endemic area for the disease [6, 7]. Here, we present successful management of a huge pulmonary hydatid cyst with lung-preserving surgery.

## 2. Case Presentation

**Figure 1:**
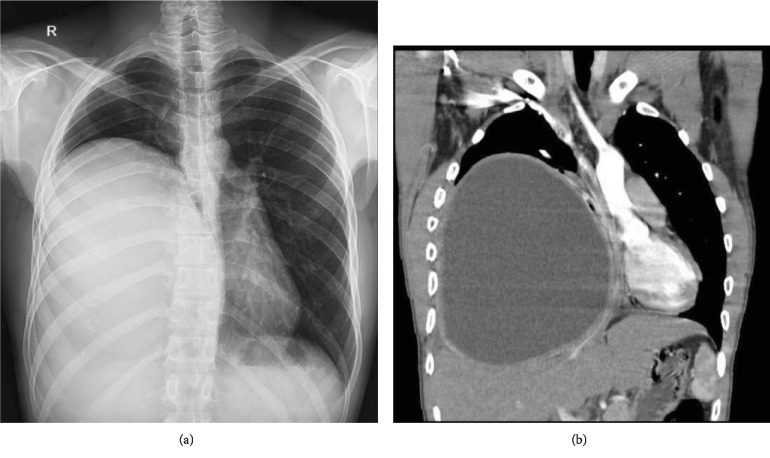
(a) Chest X-ray and (b) computed tomography (coronal view) on admission revealing a large pulmonary hydatid cyst in the right lower lobe.

**Figure 2:**
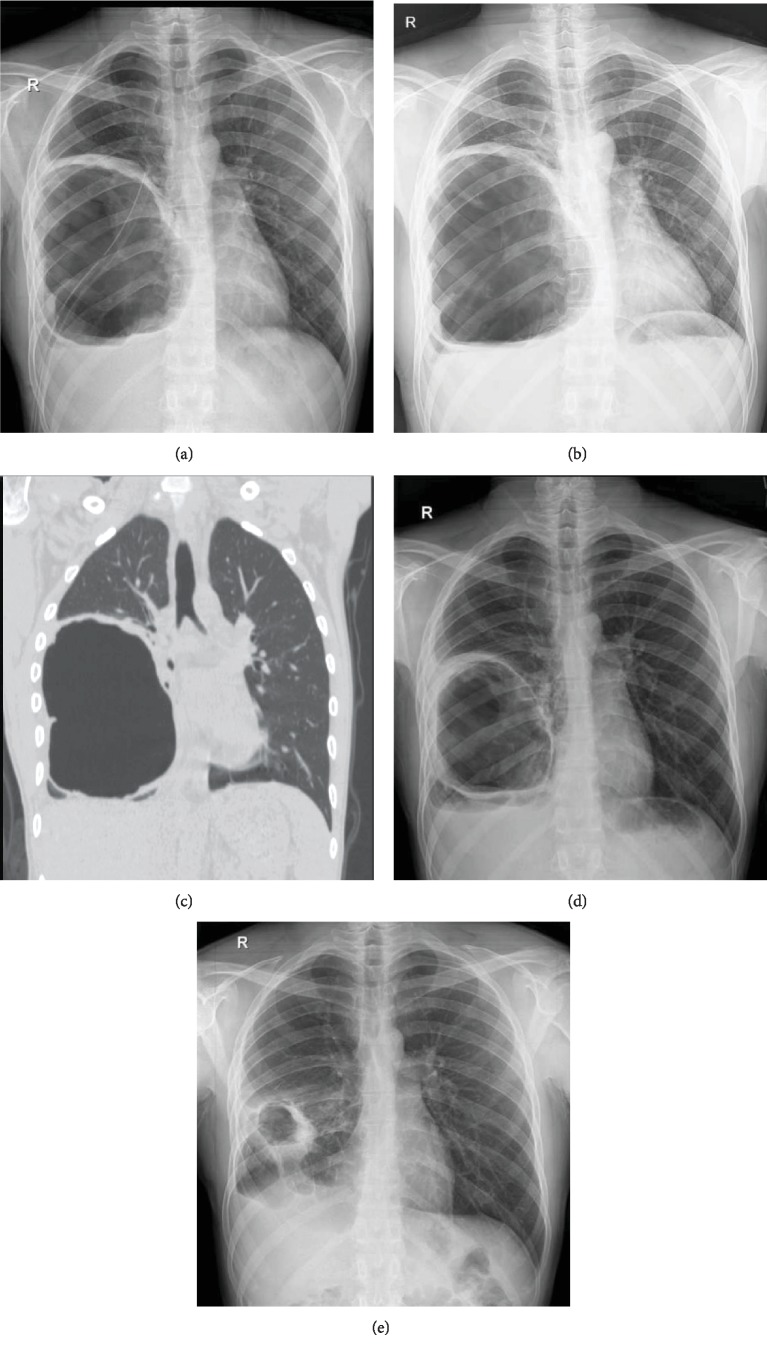
Postoperation radiography of a pulmonary hydatid cyst with lung-preserving surgery: (a) postop; (b) on discharge; (c) chest CT 2 months after surgery; (d) after a 6-month follow up; (e) after a 12-month follow up.

A 28-year-old man with no past medical and family history presented with dyspnea during strenuous activities. The radiologic finding was in favor of a large cyst (20 × 18 × 14 cm) in the right hemithorax (Figures 1(a) and 1(b)). He denied any drug or substance abuse. On physical examination, there was a decreased breathing sound in the right hemithorax and laboratory data were unremarkable. A computed tomography scan showed no evidence of a hydatid cyst, except the one mentioned above, in other locations or organs. On the day of surgery, fiberoptic bronchoscopy was done before placement of double-lumen ETT, which only showed narrowing in the orifice of the right lower lobe bronchus from external compression. The patient underwent right posterolateral thoracotomy through the 6th intercostal space, in which the cyst contents including 2500 cc clear fluid and the laminated membrane of the hydatid cyst were removed. We entered a large cystic cavity in the right lower lobe, of which walls were densely adhered to the mediastinum, chest wall, and diaphragm. Since lobectomy was technically hazardous, further dissection was abandoned and multiple large bronchopleural fistulas were individually suture ligated with Vicryl 3-0 stitches. Lung ventilation was done, and significant air leaks were closed without the lower lobe being able to expand. After inserting an N: 28F chest tube in the cavity, the chest wall was closed. The operation time was 128 minutes, and total blood loss was 280 milliliters. A postop chest X-ray showed an empty cyst cavity with no evidence of the expansion of the lung in the lower hemithorax (Figure 2(a)). The postop course was uneventful, and the chest tube was removed on the 4th postop day. The patient was discharged on the 5th postop day with a chest X-ray showing no improvement in lung expansion (Figure 2(b)) and a medical therapy consisting of oral albendazole 400 mg twice daily for a duration of three months with one-week drug cessation after each month and oral levofloxacin 750 mg daily for 10 days. Follow-up visits at the clinic were done 2 weeks, 2 months, 6 months, and one year after the operation with serial chest X-rays showing gradual obliteration of the remained cavity and resolution of the right lower lobe (Figures 2(c) and 2(d)). The patient had a significant improvement of symptoms during this period.

## 3. Discussion

It is reported in literature that chest pain, cough, and dyspnea are the most common clinical symptoms of patients presenting with a pulmonary hydatid cyst; however, our patient developed only dyspnea regarding the fact that the size of the hydatid cyst was huge [4, 8]. Kuzucu et al. reported that patients having a hydatid cyst greater than 10 cm may present with productive cough and dyspnea more frequent than those having smaller pulmonary hydatid cysts [8].

In the aspect of treatment, surgery provides the best option for the treatment of pulmonary hydatid cysts. The most common procedure for the management of lung hydatid cysts is Barrett/Posadas' technique (cystotomy and closure of bronchopleural fistulas with or without capitonnage) [9]. Although it is well accepted in the literature that parenchymal resections should be reserved as the last resort options, sometimes segmentectomy and even lobectomy may be inevitable [10, 11].

There is no generally accepted size to define the diameter of the cyst as “huge,” although in most studies, cysts more than 10 centimeters were regarded as “giant” or “huge” cysts [8]. In cases with huge hydatid cyst (more than 10 cm in diameter), postoperative complications are more frequent. Cases of a huge hydatid cyst presented with more prolonged air leakage and atelectasis; however, our case did not present with such complications after surgery [8]. The lobectomy rate in pulmonary hydatid surgery is reported to range from 0.5% to 45% in the literature. In a study by Karaoglanoglu et al., this rate was 13% in giant cysts [12].

Indications for parenchymal resection are giant cysts occupying the entire lobe, multiple cysts, and an unexpandable lobe after the excision of the cyst. It is believed that parenchyma-preserving procedures should be preferred in most cases because the lung parenchyma that has been compressed by the cyst is healthy and would expand postoperatively [12]. The decision for parenchymal resection is taken during the operation with an evaluation of the lung expansion after the excision of the cyst [13].

Significance in our case is encountering a huge cyst in the right lower lobe which was not amenable to lobectomy due to technical aspects. Cyst walls were densely adhered to the chest wall, diaphragm, and mediastinum, and dissection of fissures was hazardous. Even after meticulous airtight closure of bronchial openings, the lower lobe did not expand. The chest tube was removed early in the postop course due to the absence of air leakage to prevent further contamination of the cavity. As the cyst cavity collapsed gradually during the follow-up period, the lower lobe was well expanded. The learning point of this case is the importance of secure bronchial opening closure in the surgical management of pulmonary hydatid cysts to prevent space infection which may complicate the postop course. Even if the remaining lobe does not expand adequately during operation, gradual expansion in the long term would be expected while there is no air leakage from it.

## 4. Conclusion

Thoracic surgeons should be aware of unexpected difficulties in operating huge pulmonary hydatid cysts. Parenchyma-preserving surgery is advised and is considered fundamental in the surgical management of lung hydatidosis, and radical surgery can be avoided even in cases with a large hydatid cyst. Secure airtight closure of bronchial openings is invaluable in attaining such excellent results.
